# Biological Characterization and Metabolic Variations among Cell-Free Supernatants Produced by Selected Plant-Based Lactic Acid Bacteria

**DOI:** 10.3390/metabo13070849

**Published:** 2023-07-13

**Authors:** Wasim S. M. Qadi, Ahmed Mediani, Zalifah Mohd Kasim, Norazlan Mohmad Misnan, Norrakiah Abdullah Sani, Nur Hidayah Jamar

**Affiliations:** 1 Department of Food Science, Faculty of Science and Technology, Universiti Kebangsaan Malaysia, UKM, Bangi 43600, Malaysia; 2 Institute of Systems Biology (INBIOSIS), Universiti Kebangsaan Malaysia, UKM, Bangi 43600, Malaysia; 3 Herbal Medicine Research Centre, Institute for Medical Research, National Institutes of Health, Shah Alam 40170, Malaysia; 4 Department of Biology and Biotechnology, Faculty of Science and Technology, Universiti Kebangsaan Malaysia, UKM, Bangi 43600, Malaysia

**Keywords:** antioxidant, antibacterial, lactic acid bacteria, metabolomics, ^1^H NMR, probiotic

## Abstract

The aim of this research was to assess the antibacterial and antioxidant properties as well as the variation in metabolites of the cell-free supernatant (CFS) produced by lactic acid bacteria (LAB) from local plants: *Lactiplantibacillus plantarum* ngue16, *L. plantarum* ng10, *Enterococcus durans* w3, and *Levilactobacillus brevis* w6. The tested strains exhibited inhibitory effects against pathogens, including *Bacillus cereus*, *B. subtilis*, *Cronobacter sakazakii*, *Escherichia coli*, *Salmonella* Typhimurium, *and Staphylococcus aureus* using the agar spot assay and well diffusion method. The CFS from all four strains displayed antibacterial activity against these pathogens with minimum inhibitory concentration (MIC) values ranging from 3.12 to 12.5 mg/mL and minimal bactericidal concentration (MBC) values ranging from 6.25 to 25.0 mg/mL. Moreover, the CFS demonstrated resilience within specific pH (3–8) and temperature (60–100 °C) ranges and lost its activity when treated with enzymes, such as Proteinase K and pepsin. Furthermore, the CFS exhibited antioxidant properties as evidenced by their ability to inhibit the formation of two radicals (1,1-diphenyl-2-picrylhydrazyl (DPPH) and ferric reducing antioxidant power (FRAP) compared to the negative control, De Man, Rogosa, and Sharpe (MRS) broth. The use of proton-based nuclear magnetic resonance (^1^H-NMR) spectroscopy revealed the presence and quantification of 48 metabolites in both the CFS and MRS broths. Principal Component Analysis (PCA) effectively differentiated between CFS and MRS broth by identifying the specific metabolites responsible for the observed differences. The partial least squares (PLS) model demonstrated a significant correlation between the metabolites in the LAB supernatant and the tested antibacterial and antioxidant activities. Notably, anserine, GABA, acetic acid, lactic acid, uracil, uridine, propylene glycol, isopropanol, serine, histidine, and indol-3-lactate were identified as the compounds contributing the most to the highest antibacterial and antioxidant activities in the supernatant. These findings suggest that the LAB strains investigated have the potential to be utilized in the production of functional foods and the development of pharmaceutical products.

## 1. Introduction

Lactic acid bacteria (LAB) are a Gram-positive group of bacteria that ferment carbohydrates to produce lactic acid. They have gained attention as probiotics due to their recognized safety and ability to promote health [1]. Probiotics, according to the World Health Organization, are live microorganisms that provide beneficial effects to the host when consumed [2]. LABs qualify as probiotics because they can survive and adapt to the harsh conditions of the gastrointestinal tract, tolerate bile and acid, and adhere to intestinal cells. Moreover, they produce antimicrobial compounds that can combat pathogenic bacteria [3]. LABs not only play a vital role in food preservation and fermentation but also produce various bioactive compounds such as organic acids, hydrogen peroxide, and peptides. These compounds effectively control and inhibit the growth of pathogenic bacteria. Additionally, LAB contributes to the sensory quality of fermented products by generating aroma and flavor compounds through proteolytic activity [4]. Several studies have reported that the presence of LAB compounds in fermented foods confers health benefits regarded as being caused by bioactive compounds produced by LAB during fermentation [5,6].

In recent times, the issue of antibiotic resistance has emerged as a significant worry for both scientists and consumers, prompting the exploration of advanced solutions. The pathogenic bacterial ability to develop immunity against antibiotics has necessitated the search for alternatives, leading to studies investigating various options such as bacteriocins and organic acids [7]. To assess the efficacy of probiotics in food, a Joint FAO/WHO working group has established guidelines that specify certain desirable traits, including resistance to bile salt and low pH, susceptibility to antibiotics, and functional properties like antimicrobial and antioxidant activities [8]. Previous research has demonstrated that probiotics can effectively mitigate antibiotic resistance by not only possessing bioactive compounds but also by outcompeting harmful microorganisms for nutrients and enhancing digestive capacity. Additionally, probiotics create unfavorable conditions for pathogenic bacterial growth in the intestines by lowering the pH and acting as a protective barrier against colonization [9]. Notably, specific isolates of *Lactobacillus fermentum* obtained from *Dengke naniura* have exhibited significant antibacterial properties against the four bacteria responsible for causing diarrhea, inhibiting the growth of *Staphylococcus aureus*, *Bacillus cereus*, *Escherichia coli*, and *Salmonella* Typhi [10]. Furthermore, a previous study identified several lactic acid bacterial (LAB) strains, namely, *Levilactobacillus brevis* w6 and *E. durans* w3, isolated from vegetables, that displayed antimicrobial activity against harmful bacteria [11].

Oxygen plays a significant role in causing oxidative damage to probiotic bacteria by generating reactive oxygen species (ROS), including hydroxyl radical (OH), superoxide anion (O_2_^−^), and hydrogen peroxide (H_2_O_2_). These ROS by-products result in cell death through DNA, protein, and lipid damage [12]. To counteract ROS, most organisms possess enzymatic and non-enzymatic antioxidant agents. However, the use of synthetic antioxidants such as butylated hydroxy anisole (BHA) and butylated hydroxytoluene (BHT) is limited due to their harmful effects on the liver and potential carcinogenicity. Consequently, there has been a search for natural antioxidant substances [13]. Several studies have reported the antioxidant activity of LAB supernatant obtained from various sources [14,15,16]. LABs employ non-enzymatic components like glutathione (GSH) and thioredoxin (Trx), as well as antioxidant enzymes such as catalase, superoxide dismutase (SOD), and NADH peroxidases, to mitigate the accumulation of ROS [17]. However, the specific substances responsible for these activities remain unknown. In this study, a metabolomics approach using Proton nuclear magnetic resonance (^1^H NMR) technology was employed to identify and analyze the metabolites present in LAB strains. NMR spectroscopy has gained popularity in plant metabolomics due to its ability to detect a wide range of primary and secondary metabolites instantly. The advantages of NMR spectroscopy include fast analytical time and simple sample preparation [18]. There is limited information available on the compounds that can be extracted from LAB CFS and their correlation with antibacterial and antioxidant activities using a ^1^H NMR-based metabolomics approach. Hence, the objectives of this study were to determine the antibacterial and antioxidant activities of LAB strains derived from plant sources. The ^1^H-NMR technique was employed to identify the bioactive metabolites in the LAB supernatant. Furthermore, the study aimed to establish a relationship between antibacterial and antioxidant activities and the metabolites produced during the fermentation process.

## 2. Materials and Methods

### 2.1. Bacterial Strains and Culture Conditions

Lactic acid bacterial strains *Lactiplantibacillus plantarum* ngue16 and *L. plantarum* ng10 were isolated from *Artocarpus heterophyllus* (honey jackfruit)*. Enterococcus durans* w3 was isolated from pickled *Spondias dulcis* (Ambarella), and *Levilactobacillus brevis* w6 was isolated from pickled *Eleiodoxa conferta* (*asam kelubi*) and identified using molecular methods using 16S rRNA as stated in [11]. These strains were subjected to three rounds of proliferation in De Man, Rogosa, and Sharpe (MRS) broth (Merck^®^, Darmstadt, Germany) using a 1% inoculum in order to facilitate their growth. The incubation process took place anaerobically at 37 °C for a duration of 24 h. Six pathogenic bacteria (*Bacillus cereus* ATCC^®^33019^™^, *B. subtilis* ATCC^®^21332*^™^*, *Cronobacter sakazakii* ATCC^®^25944^™^, *Escherichia coli* O157:H7 IMR E91, *Salmonella* Typhimurium ATCC^®^14028^™^, and *Staphylococcus aureus* ATCC^®^25923^™^) were used for this study. These pathogenic strains were maintained in Muller Hinton broth (MHB) (Oxoid, Basingstoke, UK) supplemented with 40% (*v*/*v*) glycerol at a temperature of −20 °C. Prior to their utilization, the pathogens were subcultured three times using a 1% inoculum and aerobically incubated in Muller Hinton agar (MHA) (Oxoid, UK) at 37 °C with 5% CO_2_ for a period of 24 h.

### 2.2. Preparation of Cell-Free Supernatant from Lactic Acid Bacteria

Cell-free supernatants (CFS) were generated using the growth medium for LAB bacteria. Initially, the LAB cultures thrived by incubating in 10 mL of MRS broth (1% *v*/*v*) at 37 °C overnight. Subsequently, 7.5 mL of the bacterial suspension was mixed with 750 mL of MRS broth, and the mixture was incubated without shaking at 37 °C for 16 h. In order to obtain the CFS, the bacterial suspension was centrifuged at 9000 rpm for 20 min at 4 °C. The supernatant was then filtered through a 0.2-micrometer filter (Minisart^®^, Sartorius Stedim, Bohemia, NY, USA). The filtered CFS was further processed by lyophilization at a temperature below −80 °C under a pressure of less than 40 mTorr. The same preparation method was applied to the MRS broth, which served as the negative control [19].

### 2.3. Antibacterial Activity of Lactic Acid Bacterial Cell-Free Supernatant

#### 2.3.1. Spot Assay

The antibacterial potential of LAB cells was assessed against six pathogenic bacteria: three Gram-positive (*B. cereus* ATCC^®^33019^™^, *B. subtilis* ATCC^®^ 21332*^™^*, and *S. aureus* ATCC^®^25923^™^) and three Gram-negative (*E. coli* O157:H7 IMR E91, *C. sakazakii* ATCC^®^25944^™^, and *S.* Typhimurium ATCC^®^14028^™^) bacteria. The spot assay method was used to determine the antibacterial activity. Ten microliters of each LAB strain with approximately 7 log CFU/mL were placed as spots on MRS agar supplemented with 0.2% (*w*/*v*) glucose and 1.2% (*w*/*v*) agar. The plates were then incubated anaerobically at 37 °C for 24 h. Indicator bacterial suspensions of 10^6^ CFU/mL were mixed with soft MHA (0.75% agar) and poured over the MRS agar with the spot-inoculated LAB strains. The overlaid plates were incubated aerobically for 24 h at 37 °C. The experiments were performed in five replicates. The antibacterial activity was determined by measuring the diameter (in millimeters) of the growth inhibition zones surrounding each spot [20].

#### 2.3.2. Well Diffusion Assay

The antibacterial efficacy of the CFS derived from the LAB was evaluated against three Gram-positive (*B. cereus* ATCC^®^33019^™^, *B. subtilis* ATCC^®^21332*^™^*, and *S. aureus* ATCC^®^25923^™^) and three Gram-negative (*C. sakazakii* ATCC^®^25944^™^, *E. coli* O157:H7 IMR E91, and *S.* Typhimurium ATCC^®^14028^™^) bacteria. A well diffusion assay method was conducted following a previously established methodology [21]. The MHA plates were used to create circular wells with a diameter of 6 mm. These wells were then filled with 100 µL of the LAB supernatant. The pathogenic bacteria suspended in MHB at a concentration of 10^6^ CFU/mL were uniformly spread over the MHA surface using sterile cotton swabs, while MRS broth was served as the control. The inoculated MHA plates were placed in an incubator set at a temperature of 37 °C for a duration of 24 h. The zone of inhibition diameters (mm) surrounding the wells were determined to quantify the antibacterial effects of the LAB supernatant against the tested bacteria. The experiments were conducted in five replicates.

#### 2.3.3. Determination of Minimum Inhibitory Concentration and Minimal Bactericidal Concentration

The minimum inhibitory concentration (MIC) and the minimum bactericidal concentration (MBC) of LAB CFS were determined using a dilution method in 96-well polystyrene microtiter plates (Eppendorf, Germany) along with CFU counting. Initially, the CFS of each LAB strain was mixed with MHB at a concentration of 25 mg/mL and subjected to a serial dilution process with twofold increments. Subsequently, 100 µL of the diluted CFS was transferred into the wells of the 96-well plates. Simultaneously, 100 µL of fresh pathogens were added to their respective wells. The plates were then incubated at 37 °C for 24 h. The MIC endpoint was determined as the lowest concentration of CFS that exhibited no visible growth [22]. On the other hand, the MBC endpoint was defined as the lowest concentration of CFS that resulted in the elimination of over 99.9% of the pathogens, as evidenced by the absence of visible bacterial growth on the MHA plates after incubation for 24 h at 37 °C [23].

### 2.4. Characterization of Antibacterial Compounds Produced by Lactic Acid Bacterial Strains

#### 2.4.1. Effect of Heat Treatment and pH Adjustment on the Antibacterial Activity of Lactic Acid Bacterial Cell-Free Supernatant

The antibacterial efficacy of the CFS was assessed under diverse conditions to explore its potential. The impact of temperature on the CFS’s antibacterial properties was investigated by subjecting it to heat treatments at various temperatures (60, 80, 100, and 121 °C) for a duration of 30 min. Similarly, the effect of pH on the CFS’s antibacterial activity was evaluated by adjusting the pH to different levels (3, 4, 5, 6, 8, and 9) using 1 N HCl and 1 N NaOH for 30 min. Following the treatments, all the CFS samples underwent a 24-h incubation period at 37 °C using the well diffusion assay. The antibacterial effectiveness was determined by measuring the zone of inhibition surrounding the well [24].

#### 2.4.2. Effect of Enzymes on Antibacterial Activity of Lactic Acid Bacterial Cell-Free Supernatant

Proteolytic enzymes affecting the effectiveness of antibacterial activity were examined to gain insights into the active compounds involved and whether the antibacterial effect was linked to acid generation or bacteriocin synthesis. The pH of the CFS was adjusted to 6.0 using 1 N NaOH and catalase (5220 U/mg), respectively, in order to eliminate the potential effects of acid and H_2_O_2_. Subsequently, the CFS were separately treated with Proteinase K (30 U/mg) and pepsin (250 U/mg). One microliter of each enzyme was introduced into 1 mL of the CFS, followed by a one-hour incubation at ambient temperature. The enzyme-treated CFS samples were heated to 65 °C in order to halt the reaction. The CFS was assessed for its effectiveness against pathogenic bacteria using the well diffusion assay method. The CFS without any enzyme treatment served as the control for this experiment [24].

### 2.5. Antioxidant Activity of Lactic Acid Bacterial Cell-Free Supernatant

#### 2.5.1. DPPH Radical Scavenging Activity Assay

The method based on Lin and Chang [25] was used in order to measure the free radical DPPH (2,2-diphenyl-1-picrylhydrazyl) with slight modification. A 100-microliter sample of DPPH (Sigma Aldrich, Darmstadt, Germany) in ethanol (concentration of 5.9 mg/100 mL) (Sigma Aldrich, Darmstadt, Germany) was mixed with 50 µL of the CFS and MRS broth (concentration of 5 mg/mL) in a 96-well microtiter plate. After vigorously shaking the mixture, it was transferred to a dark chamber and left for 30 min at ambient temperature. Subsequently, the measurement of the absorbance at 517 nm was applied in order to calculate the scavenging activity using the given equation:

DPPH activity % = (A_517_ Control − A_517_ Sample)/A_517_ Control × 100

#### 2.5.2. Ferric Reducing Antioxidant Power (FRAP) Assay

The FRAP evaluation was performed according to the methodology described in Musa et al. [26]. A mixture containing 300 mmol/L acetate buffer (Fisher Scientific, Waltham, MA, USA) at pH 3.6, 10 mmol/L TPTZ (2, 4, 6-tripyridyl-s-triazine) (Fisher Scientific, USA) in 40 mmol/L HCl (Fisher Scientific, USA), and a 20 mmol/L FeCl_3_ 6H_2_O solution was created, with proportions of 10:1:1 for the preparation of the FRAP reagent. This resulting working reagent was utilized in the experimental procedure. Twenty microliters of the CFS and MRS broth (5 mg/mL) were combined with 200 μL of the FRAP reagent in each well of a 96-well microtiter plate. The mixture was then incubated for 45 min at ambient temperature in a dark environment. The spectrophotometer (SPEC-TRO-starNANO, BMG LabTech, Ortenberg, Germany) was used to measure the absorbance of the samples at a wavelength of 595 nm. Known concentrations of Trolox were used to establish a standard curve for comparative purposes. Linear regression analysis was used on the standard curve to determine the results, which were expressed as mmol of Trolox equivalents per gram of dry sample (mmol TE/g DW).

### 2.6. NMR Measurement and Data Pre-Processing

An investigation was conducted on the variation of metabolites in CFS using an established methodology [27]. Initially, 5 mg of freeze-dried CFS and MRS broth were combined with a DMSO-d6 solution containing 0.1% trimethylsilyl propionic acid in Eppendorf tubes (TSP). The resulting mixture was vigorously mixed for 1 min and subjected to ultrasonication for 15 min at ambient temperature. Following the centrifugation of the mixture at 13,000 rpm for 10 min, a CFS volume of 550 µL was transferred to an NMR tube for NMR analysis. The NMR spectra were acquired using a 500 MHz Varian INOVA NMR spectrometer operating at 499.887 MHz at a temperature of 25 °C. The pre-saturation (PRESAT) pulse sequence was applied to all samples in order to suppress water signals. Each spectrum was collected for 3.54 min with 64 scans. Additionally, J-resolved spectroscopy was utilized to capture a spectrum of the sample, which took 50 min and 18 sec. It involved 8 scans per 128 increments for the spin-spin coupling constant axis with spectral widths of 66 Hz and 8 K for the chemical shift axis with spectral widths of 5000 Hz, while utilizing a relaxation delay of 1.5 s. Chenomx software (version 8.2, Edmonton, Canada) was used for automated phase adjustments and baseline corrections on all sample spectra to ensure accurate data processing. Moreover, the ^1^H NMR spectra were binned using Chenomx software with consistent parameters (0.04 spectral bin) within a range of 0.5 to 10.0 ppm. The chemical shift range of 4.00–5.0 ppm, corresponding to the water signal, was excluded, resulting in a total of 222 chemical shift variables generated for each of the ^1^H NMR spectra.

### 2.7. Statistical Analyses

The experiments were performed in five replicates (n = 5), and the data obtained were then represented as the mean value along with the standard deviation. A one-way analysis of variance (ANOVA) was conducted using Minitab version 17 in order to evaluate the statistical significance. Subsequently, the Tukey’s test was applied to determine significant differences between the means, with a significance level of *p* < 0.05. After categorizing NMR spectra using Chenomx, multivariate data analysis (MVDA) was carried out using principal component analysis (PCA) and partial least squares (PLS) regression with the Parreto scaling method. This analysis was performed using SIMCA-P software (v. 14.0, Umetrics, Umeå, Sweden). In the resulting data matrix, NMR chemical shifts were treated as variables, while sample names were considered observations. A heat map and Pearson test were executed using MetaboAnalyst 5.0 in order to examine the correlation among all metabolites and identify significant metabolites. It is an online metabolomics analysis software freely accessible at http://www.metaboanalyst.ca (accessed on 15 September 2022).

## 3. Results and Discussion

### 3.1. Antibacterial Activity of LAB Cells and Cell-Free Supernatant

#### 3.1.1. Spot Assay and Well Diffusion Method

**Figure 1:**
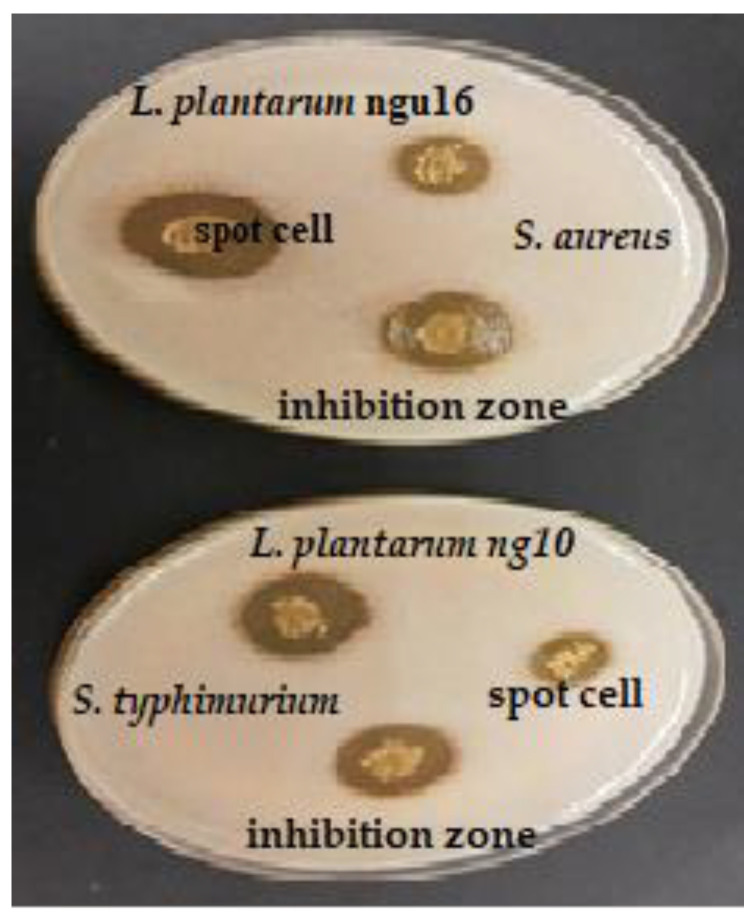
Antibacterial activity of selected lactic acid bacteria against pathogenic bacteria using the spot assay method.

The antibacterial activities of cell LAB strains were evaluated in this study, as tabulated in Table 1, and the zone inhibition around the well was measured to describe the antibacterial activity of LAB (Figure 1). All the LAB strains showed different inhibitory activities against three Gram-positive bacteria (*B. cereus* ATCC^®^33019^™^, *B. subtilis* ATCC^®^21332*^™^*, and *S. aureus* ATCC^®^25923^™^) and three Gram-negative bacteria (*C. sakazakii* ATCC^®^25944^™^, *E. coli* O157:H7 IMR E91, and *S.* Typhimurium ATCC^®^14028^™^). The diameters of the inhibition zones varied between 12.00 ± 1.00–21.62 ± 2.08 mm. The highest antibacterial activity was recorded by *L. bervise* w6 against *S. aureus* (inhibition zone: 21.6 mm), followed by *L. plantarum* ngue16 (inhibition zone: 21.0 mm). The same result was obtained by *E. durans* w3 and *L. bervise* w6 with inhibition zones of 16.0 and 16.3 mm, respectively. Inhibition zones of LAB strains varied between 12.3–17.0, 12.0–20.3, and 15.3–19.6 mm against *B. cereus*, *C. sakazakii,* and *E. coli,* respectively. *L. plantarum* ng10 and *L. plantarum* ngue16 exhibited strong activity against *B. subtilis* and *S.* Typhimurium with inhibition zones of 20.6 and 21.0 mm, respectively.

**Figure 2:**
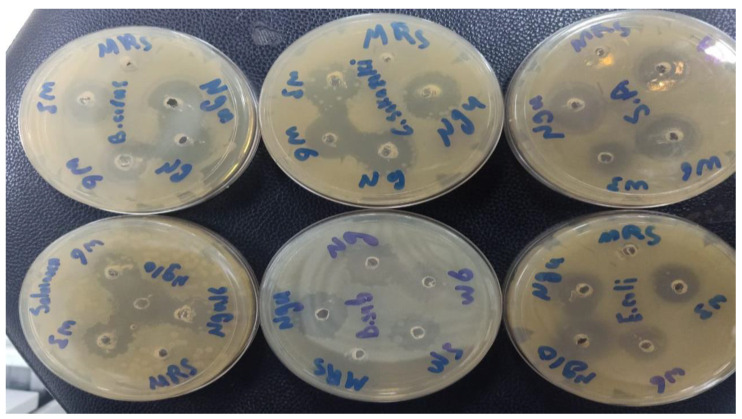
Antibacterial activity of lactic acid bacterial cell-free supernatants against pathogenic bacteria using the well diffusion method.

This study evaluated the antibacterial activities of LAB supernatant using the well diffusion method (Table 2). The inhibition zone around the well was measured to describe the antibacterial activity of LAB (Figure 2). The LAB strains had different antibacterial activities. The diameters of the inhibition zones varied between 6.8 ± 0.60–15.8 ± 0.35 mm. The highest antibacterial activity against *B. subtilis* recorded by *L. plantarum* ng10 (inhibition zone: 14.6 mm), *E. durans* w3, *L. bervise* w6, and *L. plantarum* ngue16 exhibited a good capacity for inhibiting *B. subtilis* with inhibition zones of 10.3, 9.0, and 10.6 mm, respectively. On the other hand, *L. plantarum* ngue16 exhibited the highest antibacterial activity toward *S.* Typhimurium (inhibition zone: 14.4 mm). At the same time, *L. bervise* w6 showed weak antibacterial activity against *S.* Typhimurium with an inhibition zone of 6.76 mm. In addition, *L. plantarum* ng10 showed potent antibacterial activity against *C. sakazakii* (inhibition zone: 15.8 mm). The lowest antibacterial activity toward *C. sakazakii* was recorded by *L. plantarum* ngue16, with an inhibition zone of 9.7 mm. *E. durans* w3, *L. bervise* w6, and *L. plantarum* ng10 and *L. plantarum* ngue16 exhibited close results against *E. coli* (inhibition zone diameters were 11.0, 10.3, 11.4, and 10.6 mm, respectively). *E. durans* w3 showed the highest antibacterial activity against *B. cereus* with an inhibition zone of 14.3 mm, while the lowest antibacterial activity against *B. cereus* with an inhibition zone of 7.5 mm was recorded by *L. plantarum* ng10. However, *S. aureus* was strongly inhibited by *L. plantarum* ngue16, with an inhibition zone diameter of 14.3 mm.

The antibacterial activity of LAB strains was evaluated using spot assays and well diffusion methods. In our results, the diameters of the inhibition zones varied between the two methods. There was a different trend between both methods. However, both techniques showed that all strains exhibited antibacterial activity. This difference can be attributed to the disparity in the rate of diffusion observed in each method. In the spot assay, the LAB cell is directly applied to the agar surface, allowing for immediate diffusion. As a result, the substance quickly spreads radially, forming a circular zone of inhibition. Conversely, in the well diffusion method, the substance diffuses from the wells, resulting in a slower and more controlled diffusion process. This disparity in diffusion rates can significantly influence the extent of bacterial growth inhibition, consequently affecting the measured zone sizes. Furthermore, the interaction between the substance and the agar also differs between the two methods. In the spot assay, the substance comes into direct contact with the agar surface, while in the well diffusion method, it interacts with the agar through the walls of the wells. This variation in agar interaction can further contribute to the difference in the observed results [28].

Probiotics need to possess antibacterial activity as one of their essential functions. In this study, a selection of representative pathogenic bacteria was chosen as an indicator to evaluate the antibacterial capabilities of various strains. Through spot assay and well diffusion methods, all strains demonstrated distinct antibacterial activity against the indicator organisms, confirming previous findings. The LAB-isolated Malaysian pickled mango strains, specifically *L. fermentum*, *L. pentosus*, and *L. paracasei,* exhibited antibacterial activity against ten commonly encountered foodborne bacterial pathogens. This activity was observed using LAB cultures and CFS, employing the well diffusion and spot assay methods [29]. *L. acidophilus*, isolated from honey samples, displayed antibacterial activity against multiple antibiotic-resistant *S. aureus* strains, with an inhibition zone ranging from 25.0 to 32.0 mm. It also exhibited activity against *S. epidermidis*, with an inhibition zone ranging from 14.0 to 22.0 mm, and *B. subtilis*, with an inhibition zone ranging from 12.0 to 19.0 mm, as determined by the spot assay method [30]. Various strains, including *L. plantarum*, *L. paracasei*, *E. faecium*, *L. helveticus*, *Weissella paramesenteroides*, and *Pediococcus pentosaceus*, isolated from traditional artisanal milk cheese, demonstrated antibacterial activity against *S. aureus*, *Listeria monocytogenes*, *Pseudomonas aeruginosa*, *S.* Typhimurium, and *E. faecalis*, employing the agar well diffusion assay [31]. Similarly, Sirichokchatchawan et al. [32] reported the potent antibacterial activity of *L. plantarum* 22F and 25F against pathogenic bacteria such as *E. coli*, *S. choleraesuis*, and *Streptococcus suis*. LAB strains isolated from goat’s milk and Egyptian traditional fermented milk products exhibited antibacterial activity, with varying ranges of inhibition zones spanning from 8 to 25 mm and extending from 10 to 60 mm, respectively, against indicator pathogenic organisms [33,34]. The inhibition zones observed in this research for LAB supernatant isolates against pathogens ranged from 6.76 to 15.8 mm, which were greater than the inhibition zones of 8.0 to 10.0 mm and 7.0 to 9.0 mm reported in previous studies [35].

The LAB produces a range of antimicrobial compounds, such as lactic acid, reuterin, diacetyl, hydrogen peroxide, acetic acid, phenolic compounds, benzoic acid, and bacteriocins [3,36]. Cui et al. [37] detected 12 LAB strains isolated from milk cheese in Northeast China that exhibited antibacterial activity against *S. aureus* and *E. coli*. *L. animalis*, *L. rhamnosus*, *L. fermentum*, and *L. reuteri*, isolated from fermented vegetables, demonstrated antibacterial activity against both Gram-positive and Gram-negative foodborne pathogens [38]. The current findings indicate that the ability of these four LAB strains to inhibit growth is linked to their production of organic acids, including lactic acid and acetic acid [3]. Furthermore, LAB produces peptides and bacteriocins that possess antimicrobial properties against pathogenic and non-pathogenic bacteria [39,40] demonstrated that LAB isolated from Lithuanian rye sourdoughs could serve as a natural preservative in food production due to their ability to produce organic acids and bacteriocin-like inhibitory substances (BLIS). The outcome of our study reveals that our strains have been acknowledged as promising probiotic strains with extensive applications in the food industry, functional food development, and the management of gastrointestinal disorders.

#### 3.1.2. Minimum Inhibitory Concentration and Minimal Bactericidal Concentration

Furthermore, we proceeded to determine the MIC and MBC of lyophilized CFS (concentration range: 25 mg/mL to 0.78 mg/mL, diluted in two-fold serial dilutions). The results presented in Table 3 revealed that the MIC and MBC values of the CFS derived from LAB strains against the tested foodborne pathogens fell within the range of 3.12–12.5 mg/mL and 6.25–25.00 mg/mL for MIC and MBC, respectively. Notably, ngue16 demonstrated the lowest MIC (3.12 mg/mL) and MBC (6.25 mg/mL) against *B. subtilis*, while ng10 exhibited a MIC of 3.12 mg/mL and a MBC of 6.25 mg/mL against *S.* Typhimurium. Regarding *C. sakazakii*, the MIC values for w3, w6, ng10, and ngue16 were 6.25, 3.12, 6.25, and 12.50 mg/mL, respectively. On the other hand, the MBC values for w3, w6, ng10, and ngue16 against *C. sakazakii* were 12.5, 12.5, 12.5, and 25 mg/mL, respectively. Moving on to *E. coli*, the MIC and MBC of w3 and ng10 were 3.12 mg/mL and 6.25 mg/mL, respectively, while for w6 and ngue16, they were 6.25 mg/mL MIC and 12.5 mg/mL MBC. In the case of *B. cereus*, w3, ng10, and ngue16 all recorded identical MIC and MBC values of 6.25 and 12.50 mg/mL, respectively, whereas w6 exhibited a MIC and MBC of 12.50 and 25.00 mg/mL, respectively. Lastly, the lowest MIC and MBC against *S. aureus* were observed with w6, measuring 3.12 and 6.25 mg/mL, respectively. Interestingly, w3, ng10, and ngue16 demonstrated the same MIC and MBC values against *S. aureus*, which were 6.25 and 12.5 mg/mL, respectively.

In a study, it was reported that the CFS derived from *P. pentosaceus* 4l1, isolated from fish water, displayed antibacterial activity against various strains, including *S. aureus* KCTC-1621 (MIC: 500 µg), *L. monocytogenes* KCTC-3569 (MIC: 500 µg), *B. subtilis* KCTC-3569 (MIC: 500 µg), *E. coli* O157:H7 (MIC: 500 µg), and *S. choleraesuis* ATCC^®^4731^TM^ (MIC: 300 µg) [41]. The MBC values of *P. pentosaceus* against *S. aureus* KCTC-1621, *L. monocytogenes* KCTC-3569, *B. subtilis* KCTC-3569, *E. coli* O157:H7, and *S. choleraesuis* ATCC^®^4731^™^ ranged from 500 to 1000 µg. These findings align with a previous study [42]. LAB strains identified as *P. pentosaceus* (TC48) and *L. brevis* (TC50) were isolated from fermented triticale silage. *P. pentosaceus* (TC48) exhibited MIC and MBC against *E. faecalis* (5 and 10 mg/mL), *E. coli* (5 and 10 mg/mL), *P. aeruginosa* (10 and 20 mg/mL), and *S. aureus* (10 and 20 mg/mL), respectively. *L. brevi* (TC50) exhibited the same MIC and MBC of *P. pentosaceus* (TC48) against *E. faecalis*, *E. coli and S. aureus except against P. aeruginosa* with 5 and 10 mg/mL. Moreover, a range of 0.10 to 0.30 µg/µL was observed for the MIC values, while the MBC values ranged from 0.20 to 0.50 µg/µL for LAB strains isolated from Ethiopian dairy products, targeting six food spoilage and pathogenic bacteria, including *B. cereus*, *E. coli*, *L. monocytogenes*, *P. aeruginosa*, *S. aureus,* and *S. choleraesuis* [43].

### 3.2. Characterization of Antibacterial Compounds Produced by Lactic Acid Bacterial Strains

#### 3.2.1. Effect of pH Adjustment and Heat Treatment on the Antibacterial Activity of Lactic Acid Bacterial Cell-Free Supernatant

The antibacterial activity of the LAB’s CFS is influenced by different pH levels. The CFS demonstrated stable antibacterial activity against pathogenic bacteria within a broad pH range of 3.0 to 6.0, as indicated in Table 4. The antibacterial activity of the CFS remained intact at pH 8 for 30 min, but when exposed to alkaline conditions (pH 9), the inhibitory activity against all strains was completely lost. However, the best inhibitory activity was observed at pH 3.0. This finding aligns with the stability of antibacterial compounds produced by *L. plantarum* strains isolated from *shmen*, which remained stable within a pH range of 2 to 6. However, the inhibitory activity was lost completely at pH 8 when tested against the indicator bacteria, *Lactococcus lactis* B8 [44]. Similar results were found by [45], where the antibacterial activity of CFS produced by *Lactobacillus* spp. isolated from Mexican *Cocido* cheese remained stable at pH range 2–8 against *S. aureus*, *E. coli*, *S.* Typhimurium, and *L. innocua*. Moreover, the CFS of LAB isolates from traditional cheese exhibited stability between pH 4 and 8 [46]. In contrast, Hernandez et al. [47] discovered the stability of LAB’s antibacterial activity over a wide pH range of 3–11. Our study, however, showed that the antibacterial activity of the CFS was lost entirely at pH 9 (an alkaline condition), which contradicts the previous study [36]. This suggests that the primary antibacterial activity of most strains is dependent on an acidic environment. Aween et al. [30] reported that LAB isolated from honey produced an antibacterial bacteriocin that remained stable at pH 3. However, bacteriocin ALP57 produced by *P. pentosaceus* lost its antimicrobial activity at pH 12 [48]. These observations indicate that LAB strains could be utilized as natural bio-preservative agents in milk and milk products within the pH range of 2 to 6. Notably, their remarkable effectiveness has been demonstrated in the production of low-acidic foods, including fermented milk products. 

**Figure 3:**
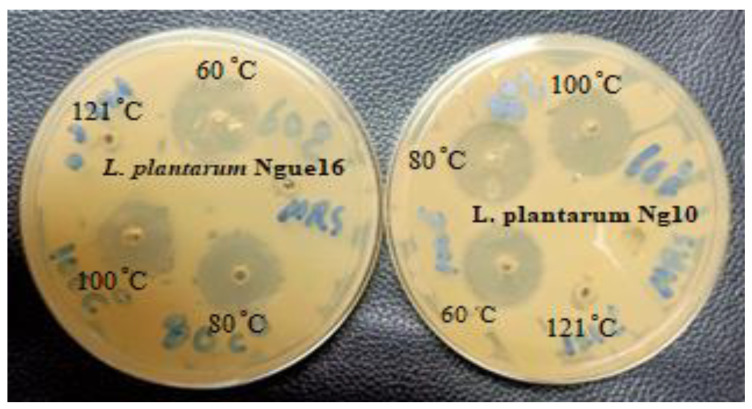
Antibacterial activity of cell-free supernatant of lactic acid bacteria at different temperatures.

The impact of temperature on the antibacterial activity of the CFS against target microorganisms is presented in Table 5 and Figure 3. The antibacterial efficacy of the CFS remained unaffected by low and moderate temperatures. However, heat sterilization at 121 °C had some effect. In general, the antibacterial activity remained stable at 100 °C for 30 min, but it became highly unstable and completely lost after exposure to 121 °C for 15 min. Similar findings were reported by [49], where antibacterial substances produced by LAB isolated from traditional Indian fermented food remained stable during heat treatment at 30, 50, and 80 °C for 30 min but lost their activity when autoclaved. Furthermore, Luo et al. [50] noted that LAB strains from kurut exhibited high stability to heat treatment, retaining their antimicrobial activity after 20 min at 100 °C, and some strains maintained their activity even after 20 min at 121 °C. Conversely, LAB strains isolated from authentic Bulgarian dairy products produced bacteriocin with high thermostability after 60 min of heat treatment at 100 °C. Additionally, most antimicrobial compounds demonstrated stability at high temperatures [51]. Khochamit et al. [52] suggested that the low molecular weight and secondary structure of antimicrobial bacteriocins contribute to their resistance against high temperatures. The discovery suggests that LAB’s antibacterial substances have the potential to serve as natural preservatives for food once it undergoes pasteurization.

#### 3.2.2. Effect of Enzymes on the Antibacterial Activity of Lactic Acid Bacterial Cell-Free Supernatant

**Figure 4:**
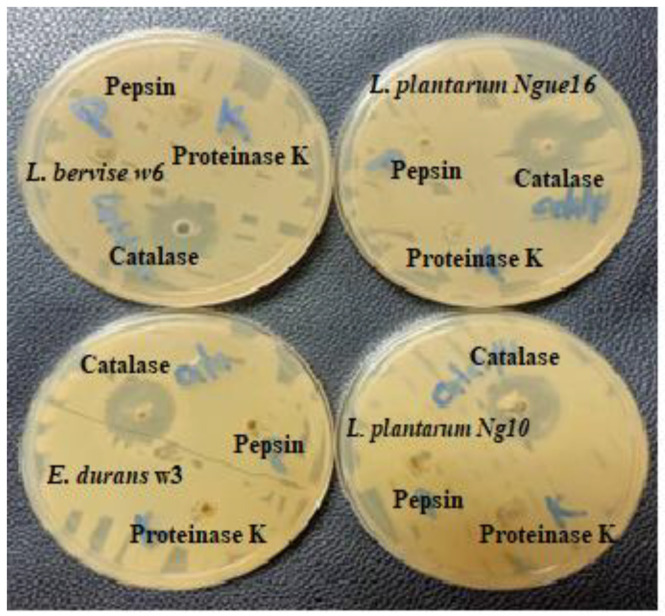
Effect of enzyme treatment on the antibacterial activity of cell-free supernatant of lactic acid bacteria.

The use of proteinase K and pepsin enzymes in the LAB supernatants resulted in a complete loss of inhibitory activity against indicator bacteria in the treated supernatants compared to the untreated supernatants. The decline in activity was attributed to the hydrolysis of antibacterial peptides present in the supernatants (Table 6 and Figure 4). Moreover, the analysis confirming the proteinaceous nature of the antibacterial compounds revealed that the LAB-produced substances resembled bacteriocins (bacteriocin-like substances, or BLS). This finding aligns with a previous study [53], which demonstrated that proteolytic enzymes such as trypsin, protease E, and proteinase K inactivated the antibacterial compounds produced by LAB strains (specifically *L. curvatus*, *L. delbrueckii*, *L. fermentum*, *E. faecium*, and *P. acidilactici*). In the case of LAB isolated from Black Sea mussels (identified as *Sporolactobacillus kofuensis*, *L. sakei*, *S. gallolyticus* ss *gallolyticus*, and *L. brevis*), their antibacterial activity against target bacteria was lost after treatment with proteinase K and trypsin, suggesting that these LAB strains were capable of producing antibacterial peptides [54]. Previous studies have also reported the presence of low molecular peptides in LAB culture supernatants following enzyme treatment [55,56]. Therefore, these findings indicate that LAB can produce antibacterial peptides once acid and catalase are removed. However, it should be noted that the peptides produced by LAB in fermented food are degraded in the intestinal tract without affecting the intestinal microflora.

### 3.3. Antioxidant Activity of LAB Cell-Free Supernatant

The occurrence of oxidative free radicals or reactive oxygen species during metabolic processes initiates the oxidation of lipids and proteins, resulting in detrimental DNA damage and progressive cellular deterioration [57]. The antioxidant effects of probiotic strains were evaluated by analyzing their DPPH and FRAP radical-scavenging activities. The results indicate that all strains’ cell-free supernatants (CFSs) effectively inhibit the formation of DPPH and FRAP radicals compared to the standard MRS broth (Table 7). Among the strains, ng10 exhibited the highest 7PPH radical-scavenging activity (79.3%), followed by ngue16, w6, and w3, with DPPH radical-scavenging activities of 76.5%, 72.6%, and 71%, respectively. On the other hand, w3 demonstrated the highest FRAP activity (80.6 mmol TE/g), with w6, ng10, and ngue16 showing comparable results (63.6, 64, and 69.6 mmol TE/g, respectively). Furthermore, the DPPH and FRAP scavenging activities of CFSs were higher than those of the MRS broth, aligning with the findings of a previous study [58]. Notably, *L. rhamnosus* CCFM 1107 exhibited greater DPPH radical scavenging capability than the MRS broth. Additionally, previous research highlighted the superior free radical scavenging activities of supernatants from various LAB strains (*L. plantarum*, *L. paracasei*, *E. faecium, L. helveticus, W. paramesenteroides,* and *P. pentosaceus*) compared to the control strain *L. rhamnosus* GG [32]. Another study conducted demonstrated that the probiotic strain *L. plantarum* 15 exhibited a DPPH scavenging activity of 75.21% and displayed different levels of reducing power (FRAP) [59]. Similarly, Han et al. [60] demonstrated that the CFS of *L. acidophilus*, *L. plantarum*, *L. curvatus, L. sake*, *P. pentosaceus*, and *L. fermentum* isolated from Harbin dry sausages possessed reducing activities exceeding 1.4 Mm. Furthermore, Das and Goyal [61] provided evidence that CFS contains intracellular antioxidants and proteins, contributing to the high reducing power of LAB. Moreover, antioxidant enzymes such as SOD, catalase, GSH S-transferase, GSH peroxidase, pseudocatalase, NADH-oxidase, and NADH peroxidase are recognized as crucial enzymatic defense systems against oxidative stress in LAB cell-free extracts [62,63].

### 3.4. Bioactive Metabolites of LAB Cell-Free Supernatant

**Figure 5:**
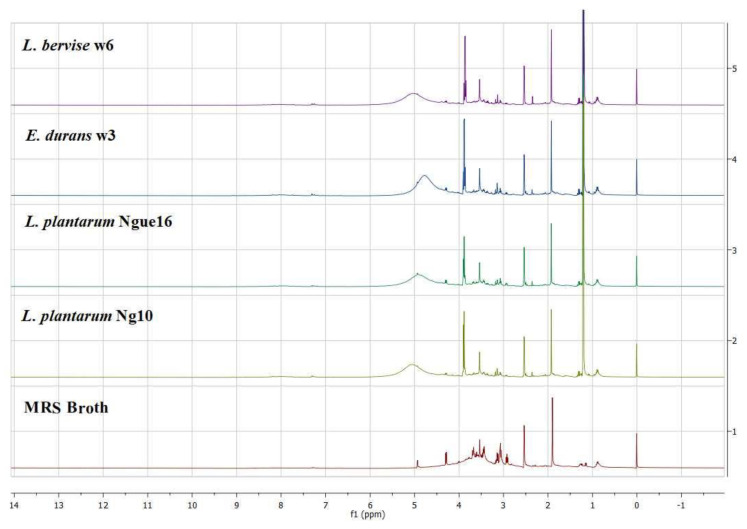
Representative ^1^H NMR spectra of lactic acid bacterial cell-free supernatant and MRS broth.

A diverse range of metabolites was found in the supernatant of LAB and MRS broth, showcasing an extensive assortment of detected components encompassing amino acids, carbohydrates, organic compounds, as well as nucleosides and nucleotides (Table 8 and Figure 5). The investigation into the composition of metabolites in the LAB supernatant and MRS broth involved multivariate data analysis (MvDA). A PCA was employed in order to gain insights into sample clustering and the metabolites contributing to the observed variations. The score plot (PCA) effectively depicted the group’s clustering patterns, while the loading plot revealed the specific metabolites responsible for the differences among the samples.

**Figure 6:**
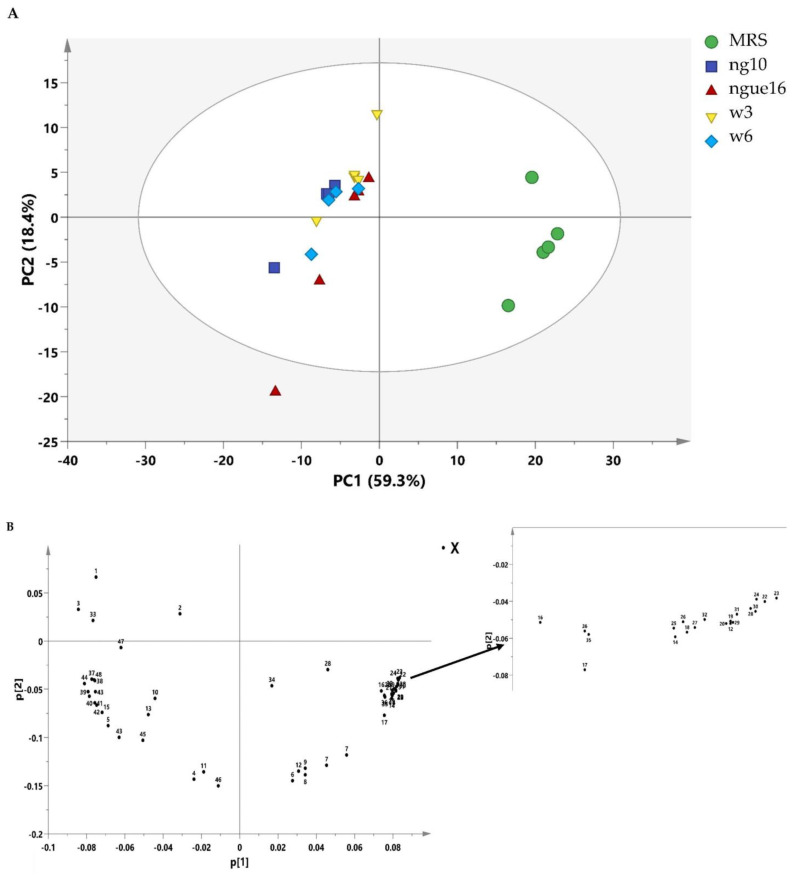
PCA score plot of lactic acid bacterial cell-free supernatant and MRS broth (**A**) and the loading plot of lactic acid bacterial cell-free supernatant and MRS broth (**B**). ngue16: *Lactiplantibacillus plantarum*; ng10: *Lactiplantibacillus plantarum*; w3: *Enterococcus durans*; w6: *Levilactobacillus brevis*. GABA: 1; Propylene Glycol: 2; Isopropanol: 3; 2-Hydroxyvalerate: 4; Lactic acid: 5; Threonine: 6; Acetoin: 7; Alanine: 8; Citrulline: 9; Acetic acid: 10; Glutamate: 11; Trans-4-hydroxy-l-proline: 12; 4-Carboxyglutamate: 13; Pyruvate: 14; Glutamine: 15; Guanidinosuccinic: 16; Isocitrate: 17; 5.6-Dihydrothymine: 18; Asparagine: 19; 3-Aminoisobutyrate: 20; Arginine: 21; Betaine: 22; Tryptophan: 23; Cellobiose: 24; Ribose: 25; Glucuronate: 26; Valine: 27; Maltose: 28; Isoleucine: 29; Fructose: 30; Galactonate: 31; 5-hydroxylysine: 32; Serine: 33; Glucose: 34; Xylose: 35; Cysteine: 36; Mannose: 37; Sucrose: 38; Uracil: 39; Uridine: 40; Allantoin: 41; NADP+: 42; Anserine: 43; Histidine: 44; Histamine: 45; Thymine: 46; Indol-3-lactate: 47; Formate: 48.

**Figure 7:**
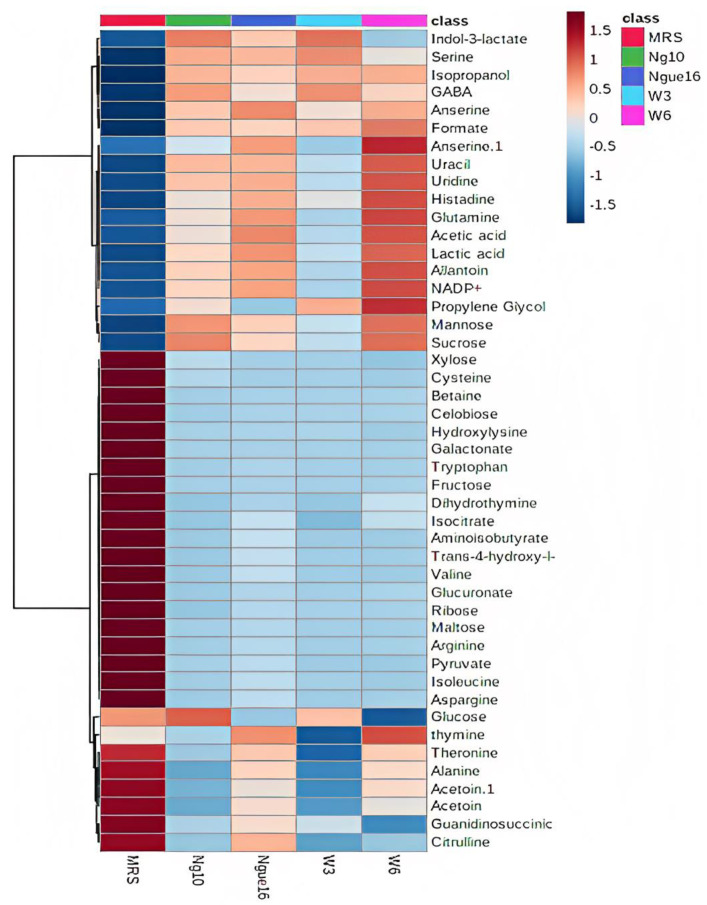
Heatmap displaying variances in metabolites between the lactic acid bacterial cell-free supernatant and MRS broth. ngue16: *Lactiplantibacillus plantarum*; ng10: *L. plantarum*; w3: *Enterococcus durans*; w6: *Levilactobacillus brevis*.

Notably, PC1 accounted for a substantial 59.3% of the total data variation, whereas PC2 explained an additional 18.4% (Figure 6A). Remarkably, the score plot clearly illustrated two distinct clusters, representing the supernatant and MRS broth samples, respectively. By referring to the loading plot **(**Figure 6B). The compounds that contribute to this differentiation encompass the metabolites anserine, GABA, acetic acid, format, lactic acid, sucrose, uracil, uridine, thymine, histamine, 2-hydroxyvalerte, propylene glycol, isopropanol, 4-carboxyglutamate, serine, glutamate, glutamine, allantoin, NADP+, histidine, mannose, and indol-3-lactate in the LAB supernatant. However, acetoin, pyruvate, glucose, fructose, maltose, ribose, cysteine, tryptophane, alanine, asparagine, arginine, isoleucine, 5-hydroxylysine, guanidinosuccinic, galactonate, xylose, 3-aminoisobutyrate, isocitrate, 2-phosphoglycerate, valine, betaine, threonine, trans-4-hydroxy-l-proline, citrulline, glucuronate, 5.6-dihydrothymine, and cellobiose in MRS broth. The major metabolites observed were GABA, propylene glycol, isopropanol, serine, indol-3-lactate, format, anserine, lactic acid, and acetic acid, which were higher in the supernatant due to the fermentation with LAB. Furthermore, the LAB supernatant exhibited varying concentrations of sucrose, uridine, uracil, thymine, 2-hydroxyvalerate, and various amino acids. Following fermentation, there was a decrease in the concentrations of the primary sugars, namely glucose, fructose, ribose, and maltose, present in the MRS broth. The heatmap shows the metabolites’ differences in the LAB supernatant among different strains (Figure 7). The variances are linked to the colors of the squares, with red representing a high contribution (dark red indicating the strongest) and blue representing a low contribution (dark blue indicating the weakest). The higher concentrations of indol-3-lactate and GABA were found in w3 and ng10; mannose and Sucrose in w6 and ng10; anserine, propylene glycol, lactic acid, and acetic acid with a high concentration in w6 and ngu16; serine in w3; formate, thymine, uracil, uridine, histidine, glutamine, allantoin, and NADP+ were abundant in w6. Similar metabolites were identified in the LAB’s supernatant in previous research. Bioactive metabolites were identified using Liquid chromatography-mass spectrometry (LCMS) in *L. fermentum* RC4 supernatant, namely GABA, L-lysine, isocitric acid, 3-methylthiopropionic acid (MTP), dimethyl sulfone (MSM), D-ribose, D-glucose, mesaconate, N-formyl-L-methionine, transaconitic acid, and carnosine [18]. Similarly, the antimicrobial compounds lactic acid, acetic acid, and ethanol were quantified by HPLC [64]. In addition, metabolites belonging to organic acids, nucleosides and nucleotides, amino acids and derivatives, and sugars were identified in *Lactobacillus* spp. supernatants [65].

Fermentation by LAB produces lactic acid, acetic acid, and other acids from mono- and disaccharides [66]. Some LABs produce volatile compounds such as isopropyl alcohol via metabolized acetone and propylene glycol via metabolized lactic acid during fermentation [67,68]. In addition, LAB has the ability to break down molecules such as tryptophan, resulting in the secretion of indole-3-lactic acid (ILA) [69]. LAB has the potential to hydrolyze proteins during fermentation, yielding peptides and amino acids. Peptides broken down from proteins by LAB vary in quantity and composition depending on the strain [67]. LAB proteolytic enzymes break down proteins and peptides by cleaving the terminal branch bonds, leading to the formation of bioactive dipeptides such as anserine and free amino acids such as glutamine, serine, histidine, glutamic acid, arginine, and 4-carboxyglutamate release [68], nucleobases, and nucleosides such as uracil, uridine, and thymine [70]. Moreover, LAB has the ability to convert monosodium glutamate into gamma-aminobutyric acid (GABA) through the enzyme glutamate decarboxylase (GAD), resulting in the release of beneficial substances [71].

**Figure 8:**
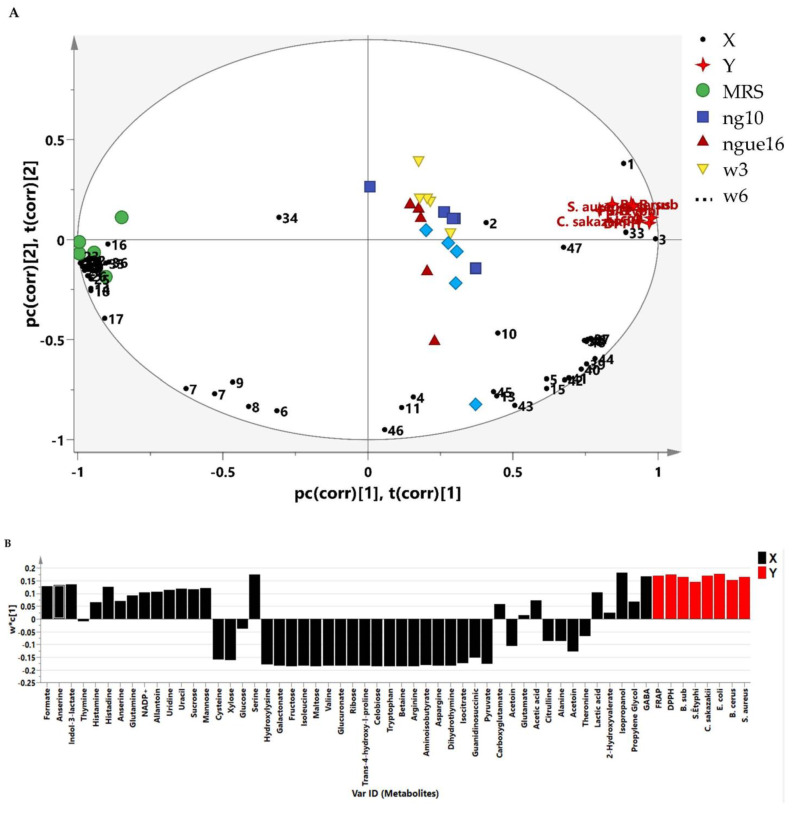
The biplot (**A**) and column plot (**B**) achieved from PLS correlate the metabolites present in the LAB supernatant with their respective antioxidant and antibacterial activities. ngue16: *Lactiplantibacillus plantarum*; ng10: *L. plantarum*; w3: *Enterococcus durans*; w6: *Levilactobacillus brevis*. GABA: 1; Propylene Glycol: 2; Isopropanol: 3; 2-Hydroxyvalerate: 4; Lactic acid: 5; Threonine: 6; Acetoin: 7; Alanine: 8; Citrulline: 9; Acetic acid: 10; Glutamate: 11; Trans-4-hydroxy-l-proline: 12; 4-Carboxyglutamate: 13; Pyruvate: 14; Glutamine: 15; Guanidinosuccinic: 16; Isocitrate: 17; 5.6-Dihydrothymine: 18; Asparagine: 19; 3-Aminoisobutyrate: 20; Arginine: 21; Betaine: 22; Tryptophan: 23; Cellobiose: 24; Ribose: 25; Glucuronate: 26; Valine: 27; Maltose: 28; Isoleucine: 29; Fructose: 30; Galactonate: 31; 5-hydroxylysine: 32; Serine: 33; Glucose: 34; Xylose: 35; Cysteine: 36; Mannose: 37; Sucrose: 38; Uracil: 39; Uridine: 40; Allantoin: 41; NADP+: 42; Anserine: 43; Histidine: 44; Histamine: 45; Thymine: 46; Indol-3-lactate: 47; Formate: 48. DPPH: 1,1-diphenyl-2-picrylhydrazyl; FRAP: ferric reducing antioxidant power.

**Figure 9:**
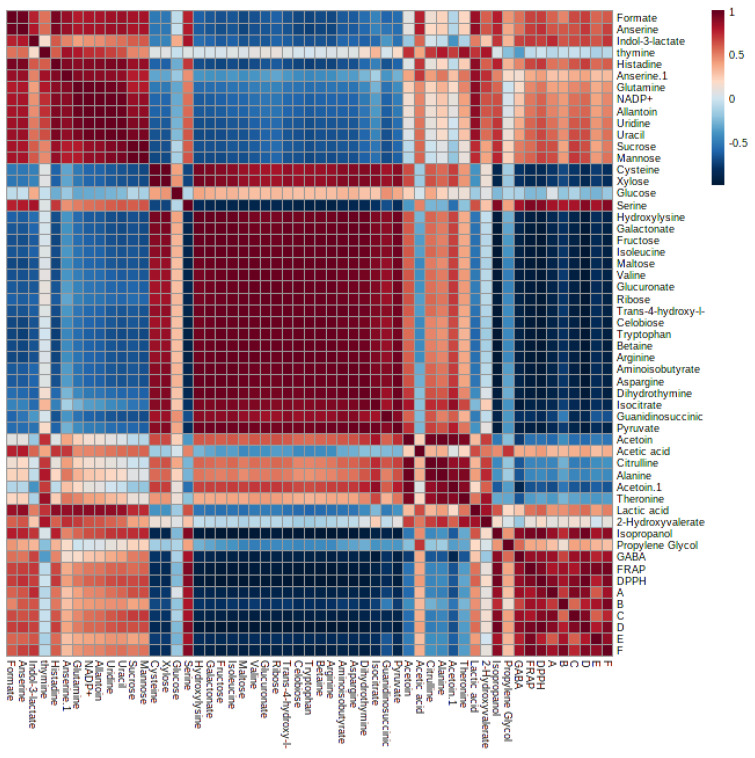
Pearson’s correlation analysis of the key metabolites identified using the Partial Least Squares (PLS) model. A: *B. subtilis*; B: *S.* Typhimurium; C: *C. sakazakii*; D: *E. coli*; E: *B. cereus*; F: *S. aureus*.

Many research investigations have been carried out to explore the biological characteristics of the supernatant of LAB. However, there is a lack of comprehensive information regarding the metabolite composition of the supernatant. Additionally, a deeper understanding of the metabolic similarities and differences among LAB strains is necessary. This research employed metabolomics to establish connections between the antibacterial and antioxidant activities of LAB supernatant and MRS broth and their respective metabolite profiles. The PLS model’s results showed a noteworthy connection between the metabolites found in the LAB supernatant and their antibacterial and antioxidant effects against different strains, including *S.* Typhimurium, *C. sakazakii*, *S. aureus*, *B. subtilis*, *B. cereus*, and *E. coli.* The PLS biplot (Figure 8A) and column plot (Figure 8B) revealed that LAB supernatant was closely associated with both antibacterial and antioxidant activities. It reveals the intricate correlation between the metabolites present in the LAB supernatant and their respective antioxidant and antibacterial activities. Notably, the most influential compounds contributing to these activities were anserine, GABA, acetic acid, lactic acid, uracil, uridine, propylene glycol, isopropanol, serine, histidine, and indol-3-lactate, which aligned with previous research. Prior research has shown that LAB can produce diverse compounds such as organic acids, alcohols, phenolics, exopolysaccharides, bacteriocins, and bioactive peptides through fermentation. These compounds exhibit antimicrobial and antioxidant properties [72]. The Pearson correlation coefficients depicted in Figure 9 supported the data obtained from PLS analysis. The PLS analysis of the key metabolites identified using the PLS model reveals the connection between the metabolites and the antioxidant and antibacterial properties of LAB’s cell-free supernatant. Based on Figure 9, the correlation coefficients are depicted by color-coded squares, where red signifies positive correlations (with darker shades indicating stronger relationships) and blue represents negative correlations (with darker shades indicating weaker relationships). The metabolites studied include DPPH and FRAP.

Notably, LAB-produced GABA exhibited potent antioxidant and antibacterial activities against *S.* Typhi DMST 22842, *B. cereus* TISTR 687, and *Shigella dysenteriae* DMST 1511, as previously reported [73]. Isopropyl alcohol (isopropanol) has also been identified as an antioxidant and antimicrobial agent. Furthermore, LAB can generate antibacterial substances such as lactic acid and acetic acid to combat phytopathogens [74]. Other studies have reported an increase in the concentration of polar amino acids, including glutamic acid, aspartic acid, serine, histidine, and cysteine, following protein fraction hydrolysis by LAB, which may contribute to enhanced HO• activity [75]. In a previous study, several bioactive metabolites, namely propylene glycol, lactic acid, acetic acid, acetoin, and GABA, were identified as having potential antibacterial effects against various pathogens [76,77].

**Figure 10:**
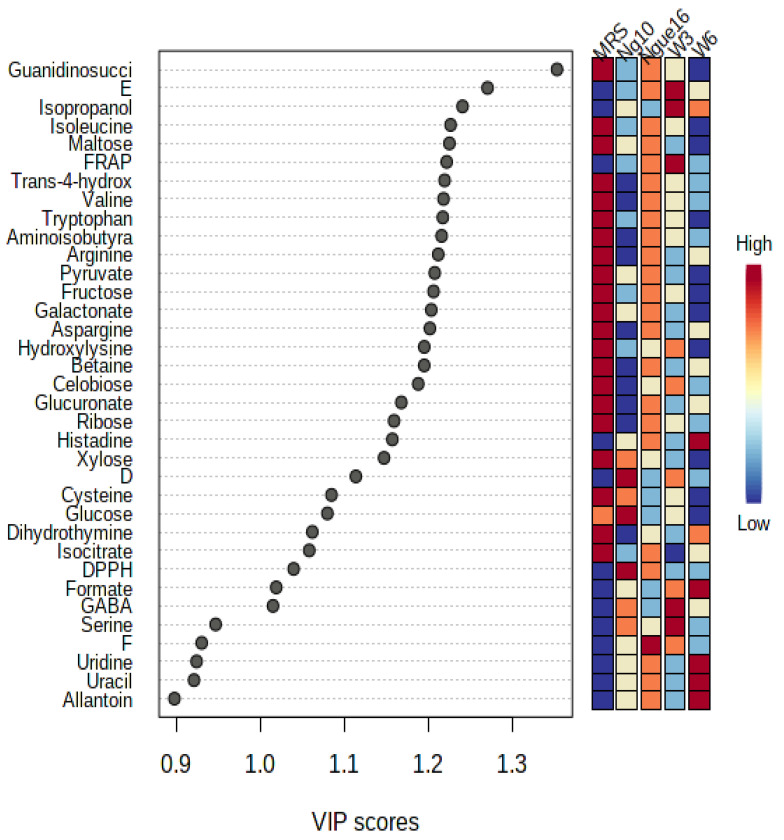
Variables important in the projection (VIP) values derived from PLS showing the significant metabolites with antioxidant and antibacterial activities in the lactic acid bacterial supernatant. ngue16: *Lactiplantibacillus plantarum*; ng10: *L. plantarum*; w3: *Enterococcus durans*; w6: *Levilactobacillus brevis*; DPPH: 1,1-diphenyl-2-picrylhydrazyl; FRAP: ferric reducing antioxidant power. D: *E. coli*; E: *B. cereus*; F: *S. aureus*.

The variable importance values in projections (VIP) were employed to identify the primary factors responsible for the biological activity. Strains ng10 and w3 demonstrated the most potent antioxidant activity using DPPH and FRAP assays, respectively. Similarly, ngue16, ng10, and w3 exhibited the strongest antibacterial activity against *S. aureus*, *E. coli,* and *B. cereus,* respectively. The VIP values depicted in Figure 10 through the PLS biplot indicate the significance of each variable in cluster separation. Variables that possess VIP values greater than 0.9 play a crucial role in the correlation and prediction of the PLS model. Consequently, these variables can be linked to chemical markers and bioactive compounds present in the supernatant of LABs, thereby indicating their significant significance in the analysis and interpretation of the model.

**Figure 11:**
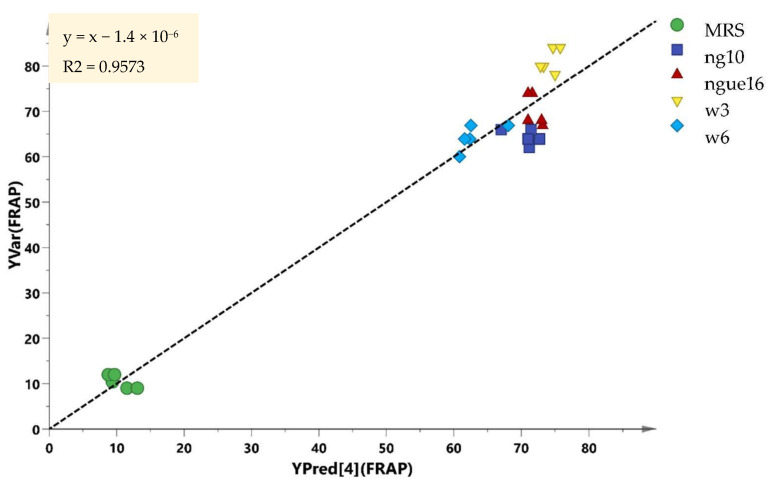
Prediction versus observation from all samples. The R2 of the regression line indicates the goodness of fit between experimental observations and the predicted model. The R2 in this correlation was 0.9573.

In this particular investigation, the Q2 and R2 values exceeded 0.8, signifying that all the models effectively validated the data and made highly accurate predictions. The PLS model’s validity was established through a robust combination of 100 permutation tests and regression validation. To ascertain and authenticate the relationship between the variables, correlation coefficients (R) were determined. In order to validate the samples, the experimental bioactivity values were derived as regression plots (Figure 11), depicting their relationship with the predicted values. These studies yielded significant results, further affirming the potency of PLS models in accurately predicting and validating the parameters of interest.

## 4. Conclusions

The results of this study indicate that specific strains of LAB obtained from plant-based sources in Malaysia exhibit advanced probiotic properties, including effective antibacterial activity against various harmful bacteria such as *B. cereus*, *B. subtilis*, *C. sakazakii*, *E. coli*, *S.* Typhimurium, and *S. aureus*. The antibacterial compounds derived from these LAB strains demonstrate resistance to a wide range of temperatures (60–100 °C) and acidity levels (pH 3–8). Furthermore, the LAB strains have demonstrated the ability to produce antibacterial peptides, as evidenced by the inactivation of the supernatant after enzyme treatment. Moreover, the LAB strains exhibit antioxidant activity, as measured by FRAP and DPPH assays. The presence of numerous bioactive compounds within the LAB supernatant is responsible for the intricate array of biological activities exhibited, such as anserine, GABA, acetic acid, lactic acid, uracil, uridine, propylene glycol, isopropanol, serine, histidine, and indol-3-lactate, which were identified using ^1^H NMR analysis. These findings highlight the potential of these selected LAB strains as valuable resources for the development of probiotics and antioxidant-enriched functional foods.

## Figures and Tables

**Table 1:** Antibacterial activity of lactic acid bacteria against target bacteria using the spot assay method.

Strain	*B. cereus*	*B. subtilis*	*C. sakazakii*	*E. coli*	*S. aureus*	*S.* Typhimurium
	Zone of Inhibition (mm)					
w3	17.00 ± 1.73 ^a^	20.00 ± 1.15 ^a^	12.00 ± 1.00 ^d^	15.32 ± 1.52 ^c^	16.00 ± 2.64 ^b^	16.00 ± 1.00 ^b^
w6	16.66 ± 1.15 ^b^	18.31 ± 2.08 ^b^	14.32 ± 1.15 ^b^	16.60 ± 1.52 ^b^	21.62 ± 2.08 ^a^	14.00 ± 1.00 ^c^
ng10	12.30 ± 0.57 ^d^	15.60 ± 2.64 ^c^	20.33 ± 1.15 ^a^	19.62 ± 1.15 ^a^	16.32 ± 2.3 ^b^	21.00 ± 1.73 ^a^
ngue16	13.63 ± 1.52 ^c^	20.62 ± 0.57 ^a^	13.60 ± 1.52 ^c^	19.64 ± 2.51 ^a^	21.00 ± 2.64 ^a^	17.00 ±2.00 ^b^

The mean values with their corresponding standard deviations are presented (n = 5). ^a–d^ Superscript letters are used to indicate significant differences within the row (*p* < 0.05). ngue16: *Lactiplantibacillus plantarum*; ng10: *L. plantarum*; w3: *Enterococcus durans*; w6: *Levilactobacillus brevis*.

**Table 2:** Antibacterial activity of lactic acid bacteria cell-free supernatant against pathogenic bacteria using the well diffusion method.

Strain	Intial pH	*B. cereus*	*B. subtilis*	*C. sakazakii*	*E. coli*	*S. aureus*	*S.* Typhimurium
		Zone of Inhibition (mm)					
w3	3.98	14.3 ± 0.57 ^a^	10.3± 0.57 ^a^	11.6 ± 1.52 ^a^	11.0 ± 0.40 ^a^	13.8 ± 0.8 ^a^	10.4 ± 0.96 ^a^
w6	4.05	9.4 ± 0.51 ^b^	9.0 ± 0.00 ^a^	12.3 ± 0.57 ^b^	10.3 ± 1.15 ^a^	10.0 ± 0.00 ^b^	6.8 ± 0.60 ^b^
ng10	4.19	7.5 ± 0.70 ^c^	14.6 ± 1.52 ^b^	15.8 ± 0.35 ^c^	11.4 ± 0.60 ^a^	12.4 ± 0.60 ^a^	8.8 ± 0.28 ^c^
ngue16	4.10	11.2 ± 0.72 ^d^	10.6 ± 1.15 ^a^	9.7 ± 0.75 ^d^	10.6 ± 0.57 ^a^	14.3 ± 1.15 ^a^	14.4 ± 0.66 ^d^

The mean values are presented along with their respective standard deviations (n = 5). ^a–d^ Significant differences among the values within the same row are indicated by different superscript letters (*p* < 0.05). ngue16: *Lactiplantibacillus plantarum*; ng10: *L. plantarum*; w3: *Enterococcus durans*; w6: *Levilactobacillus brevis.*

**Table 3:** Antibacterial activity of lactic acid bacterial cell-free supernatant by minimum inhibitory concentration and minimum bacterial concentration.

Strain	*B. cereus*		*B. subtilis*		*C. sakazakii*		*E. coli*		*S. aureus*		*S.* Typhimurium	
	MIC and MBC (mg/mL)											
	MIC	MBC	MIC	MBC	MIC	MBC	MIC	MBC	MIC	MBC	MIC	MBC
w3	6.25	12.50	6.25	12.50	6.25	12.50	3.12	6.25	6.25	12.50	6.25	12.50
w6	12.50	25.00	6.25	12.50	3.12	12.50	6.25	12.50	3.12	6.25	6.25	12.50
ng10	6.25	12.50	12.50	25.00	6.25	12.50	3.12	6.25	6.25	12.50	3.12	6.25
ngue16	6.25	12.50	3.12	6.25	12.50	25.00	6.25	12.50	6.25	12.50	6.25	12.50

MIC: Minimum inhibitory concentration; MBC: Minimal bactericidal concentration. ngue16: *Lactiplantibacillus plantarum*; ng10: *L. plantarum*; w3: *Enterococcus durans*; w6: *Levilactobacillus brevis*.

**Table 4:** Antibacterial activity of cell-free supernatant of lactic acid bacteria at different acidities using a well diffusion assay.

Strain	pH	*B. cereus*	*B. subtilis*	*C. sakazakii*	*E. coli*	*S. aureus*	*S.* Typhimurium
Zone of Inhibition (mm)							
w3	3	12.40 ± 0.52 ^Aa^	11.92 ± 0.36 ^Aa^	10.00 ± 0.40 ^Aa^	12.33 ± 0.57 ^Aa^	9.92 ± 0.25 ^Aa^	12.32 ± 0.57 ^Aa^
	4	10.63 ± 0.52 ^Ab^	9.00 ± 1.00 ^Ab^	9.42 ± 0.75 ^Ab^	11.40 ± 0.55 ^Ab^	9.30 ± 0.70 ^Aa^	11.10 ± 0.8 ^Ab^
	6	7.66 ± 0.72 ^Ac^	6.60 ± 0.61 ^Ac^	7.80 ± 0.28 ^Ac^	8.22 ± 0.68 ^Ac^	7.54 ± 0.50 ^Ac^	9.00 ± 0.57 ^Ac^
	8	4.10 ± 0.28 ^Ad^	5.52 ± 0.5 ^Ad^	5.00 ± 0.45 ^Ad^	6.33 ± 0.57 ^Ad^	6.60 ± 0.57 ^Ad^	6.12 ± 0.36 ^Ad^
	9	N.I	N.I	N.I	N.I	N.I	N.I
w6	3	10.22 ± 0.34 ^Ba^	11.82 ± 0.60 ^Aa^	13.52 ± 0.92 ^Ba^	11.62 ± 1.15 ^Ba^	9.74 ± 0.40 ^Ab^	8.74 ± 0.68 ^Bb^
	4	10.90 ± 1.00 ^Aa^	9.40 ± 0.51 ^Ab^	11.60 ± 0.57 ^Bb^	9.84 ± 0.76 ^Bb^	10.30 ± 1.15 ^Ba^	9.90 ± 0.7 ^Ba^
	6	8.64 ± 0.57 ^Bb^	8.62 ± 1.15 ^Bc^	8.50 ± 0.51 ^Bc^	8.60 ± 0.57 ^Ac^	9.32 ± 0.57 ^Bc^	7.62 ± 0.57 ^Bc^
	8	6.00 ± 0.50 ^Bc^	6.80 ± 0.76 ^Bd^	6.84 ± 0.28 ^Bd^	5.30 ± 0.57 ^Bd^	5.00 ± 0.00 ^Bd^	6.60 ± 0.30 ^Ad^
	9	N.I	N.I	N.I	N.I	N.I	N.I
ng10	3	9.00 ± 0.00 ^Ca^	11.60 ± 0.85 ^Ab^	13.62 ± 0.70 ^Ba^	10.92 ± 1.01 ^Ca^	9.62 ± 0.57 ^Aa^	10.82 ± 0.60 ^Ca^
	4	8.51 ± 0.50 ^Bb^	13.52 ± 0.50 ^Ba^	12.80 ± 0.28 ^Cb^	9.00 ± 0.00 ^Bb^	8.50 ± 0.50 ^Cb^	8.61 ± 0.57 ^Cb^
	6	6.63 ± 0.57 ^Cc^	10.20 ± 0.68 ^Cc^	10.55 ± 0.51 ^Cc^	7.42 ± 0.52 ^Bc^	7.00 ± 0.00 ^Ac^	7.40 ± 0.50 ^Bc^
	8	5.10 ± 0.28 ^Cd^	6.32 ± 0.28 ^Bd^	5.63 ± 0.57 ^Ad^	6.00 ± 0.00 ^Ad^	5.62 ± 0.57 ^Bd^	5.62 ± 0.57 ^Bd^
	9	N.I	N.I	N.I	N.I	N.I	N.I
ngue16	3	9.82 ± 0.76 ^Ca^	11.32 ±0.57 ^Aa^	10.32 ± 0.57 ^Aa^	11.13 ± 0.57 ^Ba^	9.51 ± 0.51 ^Aa^	9.50 ± 0.50 ^Da^
	4	8.94 ± 0.85 ^Bb^	9.62 ± 0.57 ^Ac^	9.31 ± 0.57 ^Ab^	10.80 ± 0.76 ^Cb^	9.32 ± 0.57 ^Aa^	9.70 ± 0.95 ^Ca^
	6	7.66 ± 0.57 ^Ac^	10.00 ± 1.00 ^Cb^	8.40 ± 0.51 ^Bc^	9.90 ± 0.40 ^Cc^	7.41 ± 0.52 ^Ab^	8.62 ± 0.69 ^Cb^
	8	5.00 ± 0.00 ^Cd^	5.82 ± 0.76 ^Ad^	5.32 ± 0.57 ^Ad^	7.00 ± 1.00 ^Cd^	6.50 ± 1.04 ^Ac^	6.30 ± 0.57 ^Ac^
	9	N.I	N.I	N.I	N.I	N.I	N.I

The mean values along with their corresponding standard deviations (n = 5) were used to express the data. ^A–D^ Different capital letters within the column represent significant differences (*p* < 0.05) among strains at the same pH. ^a–d^: Different superscript letters within the column represent significant differences (*p* < 0.05) for the same strain at different pHs. N.I: no inhibition; ngue16: *Lactiplantibacillus plantarum*; ng10: *L. plantarum*; w3: *Enterococcus durans*; w6: *Levilactobacillus brevis.*

**Table 5:** Antibacterial activity of cell-free supernatant of lactic acid bacteria at different temperatures using a well diffusion assay.

Strain	Temperature °C	*B. cereus*	*B. subtilis*	*C. sakazakii*	*E. coli*	*S. aureus*	*S.* Typhimurium
Zone of Inhibition (mm)							
w3	60	13.10 ± 0.76 ^Aa^	13.32 ± 1.15 ^Aa^	13.22 ± 0.68 ^Aa^	10.00 ± 0.50 ^Aa^	9.62 ± 0.57 ^Aa^	12.00 ± 0.00 ^Aa^
	80	9.60 ± 0.57 ^Ab^	12.30 ± 0.5 ^Ab^	10.80 ± 0.76 ^Ab^	10.33 ± 0.57 ^Ab^	10.34 ± 0.57 ^Aa^	11.62 ± 0.57 ^Ab^
	100	9.00 ± 1.00 ^Ab^	12.10 ± 0.36 ^Ab^	8.33 ± 1.15 ^Ac^	9.66 ±0.57 ^Ac^	9.80 ± 0.28 ^Aa^	10.30 ± 0.57 ^Ac^
	121	N.I	N.I	N.I	N.I	N.I	N.I
w6	60	11.63 ± 1.52 ^Ba^	11.32 ± 0.57 ^Ba^	11.66 ±0.57 ^Ba^	11.31 ± 1.15 ^Ba^	8.80 ± 0.23 ^Ba^	14.00 ±1.00 ^Ba^
	80	10.60 ± 1.15 ^Bb^	9.50 ± 0.50 ^Bb^	10.14 ± 1.01 ^Ab^	10.00 ± 1.00 ^Ab^	7.60 ± 0.57 ^Bb^	10.64 ± 1.15 ^Bb^
	100	8.62 ± 0.57 ^Bc^	6.30 ± 0.57 ^Bc^	10.32 ± 0.57 ^Bb^	7.33 ± 0.57 ^Bc^	6.82 ± 0.76 ^Bc^	9.60 ± 0.52 ^Bc^
	121	N.I	N.I	N.I	N.I	N.I	N.I
ng10	60	10.64 ± 1.52 ^Ca^	12.90 ± 0.17 ^Aa^	12.15 ±1.04 ^Ba^	9.51 ± 0.86 ^Ca^	11.32 ± 1.15 ^Ca^	12.63 ± 0.57 ^Aa^
	80	9.00 ± 1.00 ^Cb^	11.52 ± 1.3 ^Cb^	12.30 ± 0.57 ^Ba^	8.62 ± 0.57 ^Bb^	10.60 ± 0.57 ^Ab^	10.70 ± 0.68 ^Bb^
	100	9.40 ± 0.51 ^Ab^	10.55 ± 0.51 ^Cc^	9.62 ± 1.15 ^Cb^	8.80 ± 0.28 ^Cb^	10.30 ± 0.57 ^Cb^	10.10 ±0.40 ^Ab^
	121	N.I	N.I	N.I	N.I	N.I	N.I
ngue16	60	10.32 ± 1.52 ^Ca^	12.38 ± 0.57 ^Aa^	10.00 ± 0.00 ^Ca^	13.66 ± 0.57 ^Da^	10.63 ± 0.57 ^Da^	11.12 ± 0.28 ^Ca^
	80	8.60 ± 0.57 ^Db^	11.72 ± 0.64 ^Ca^	10.34 ± 1.15 ^Ab^	11.32 ± 1.5 2 ^Cb^	11.32 ± 1.15 ^Cb^	11.33 ± 0.57 ^Aa^
	100	8.62 ± 0.57 ^Bb^	10.20 ± 0.34 ^Cb^	10.30 ± 0.57 ^Bb^	9.30 ± 0.57 ^Ac^	9.60 ± 0.57 ^Ac^	10.30 ± 0.57 ^Ab^
	121	N.I	N.I	N.I	N.I	N.I	N.I

The mean values along with their corresponding standard deviations (n = 5) were used to express the data. ^A–D^ Different capital letters within the column represent significant differences (*p* < 0.05) among strains at the same temperature. ^a–c^: Different superscript letters represent significant differences within the column (*p* < 0.05) for the same strain at different temperatures. N.I: no inhibition; ngue16: *Lactiplantibacillus plantarum*; ng10: *L. plantarum*; w3: *Enterococcus durans*; w6: *Levilactobacillus brevis*.

**Table 6:** Inhibitory activity of cell-free supernatant of lactic acid bacteria following enzyme treatments using a well diffusion assay.

Strain	*B. cereus*	*B*. *subtilis*	*C. sakazakii*	*E. coli*	*S. aureus*	*S.* Typhimurium
	Zone of Inhibition (mm)					
w3						
proteinase K	N.I	N.I	N.I	N.I	N.I	N.I
pepsin	N.I	N.I	N.I	N.I	N.I	N.I
catalase	7.6 ± 0.57 ^a^	8.32 ± 1.15 ^a^	6.32 ± 0.57 ^a^	5.60 ± 0.57 ^a^	8.60 ± 0.57 ^a^	7.34 ± 1.15 ^a^
w6						
proteinase K	N.I	N.I	N.I	N.I	N.I	N.I
pepsin	N.I	N.I	N.I	N.I	N.I	N.I
catalase	7.6 ± 1.52 ^a^	7.00 ± 0.00 ^b^	6.82 ± 0.23 ^a^	6.66 ± 0.57 ^b^	6.22 ± 0.40 ^b^	8.00 ± 1.00 ^b^
ng10						
proteinase K	N.I	N.I	N.I	N.I	N.I	N.I
pepsin	N.I	N.I	N.I	N.I	N.I	N.I
catalase	6.3 ± 0.57 ^b^	7.3 ± 1.15 ^b^	8.60 ± 0.57 ^b^	7.00 ± 0.00 ^c^	6.64 ± 0.57 ^b^	6.60 ± 0.57 ^c^
ngue16						
proteinase K	N.I	N.I	N.I	N.I	N.I	N.I
pepsin	N.I	N.I	N.I	N.I	N.I	N.I
catalase	5.6 ± 0.57 ^c^	6.63 ± 0.57 ^c^	5.66 ± 0.57 ^c^	6.32 ± 1.15 ^b^	6.23 ± 0.46 ^b^	7.60 ± 1.15 ^a^

The mean values along with their corresponding standard deviations (n = 5) were used to express the data. ^a–c:^ Different superscript letters represent significant differences within the column (*p* < 0.05) among the strains. N.I: no inhibition; ngue16: *Lactiplantibacillus plantarum*; ng10: *L. plantarum*; w3: *Enterococcus durans*; w6: *Levilactobacillus brevis.*

**Table 7:** Antioxidant activity of the lactic acid bacteria cell-free supernatant as evaluated using DPPH (%) and FRAP (mmol TE/g) assays.

Strains	DPPH %	FRAP (mmol TE/g)
w3	71.0 ± 2.64 ^a^	80.6 ± 3.05 ^a^
w6	72.6 ± 2.51 ^a^	63.6 ± 3.51 ^b^
ng10	79.3 ± 1.52 ^b^	64.0 ± 2.00 ^b^
ngue16	76.5 ± 0.70 ^c^	69.6 ± 3.78 ^c^
MRS broth	33.0 ± 3.60 ^d^	10.4 ± 1.50 ^d^

The mean values are presented along with their respective standard deviations (n = 5). ^a–d^ Significant differences among the values within the same row are indicated by different superscript letters (*p* < 0.05). ngue16: *Lactiplantibacillus plantarum*; ng10: *L. plantarum*; w3: *Enterococcus durans*; w6: *Levilactobacillus brevis.*

**Table 8:** ^1^H NMR signals of metabolites identified in the cell-free supernatant and MRS broth, +/− (+: high concentration; −: low concentration).

Metabolites	Characteristic Signals	MRS Broth	Cell-Free Supernatant
GABA	0.98	−	+
Acetic acid	1.86	−	+
Acetoin	1.42, 2.22	+	−
Format	8.42	−	+
Lactic acid	1.3	−	+
Pyruvate	2.38	+	−
Glucose	3.9	+	−
Sucrose	5.22	−	+
Fructose	3.7	+	−
Maltose	3.82	+	−
Ribose	3.5	+	−
Cysteine	3.98	+	−
Uracil	5.79	−	+
Uridine	5.9	−	+
Thymine	7.34	−	+
Tryptophane	3.3	+	−
Alanine	1.5	+	−
Asparagine	2.98	+	−
Isoleucine	3.66	+	−
5-hydroxylysine	3.78	+	−
Guanidinosuccinic	2.46	+	−
Histamine	7.26	−	+
Galactonate	3.74	+	−
Xylose	3.94	+	−
2-hydroxyvalerte	1.26	−	+
Propylene glycol	1.14	−	+
3-aminoisobutyrate	3.02	+	−
Isopropanol	1.18	−	+
Isocitrate	2.62	+	−
Glutamine	2.4, 6.9	−	+
2-phosphoglycerate	3.94	+	−
4-carboxyglutamate	2.34	−	+
Anserine	7.18, 8.34	−	+
Valine	3.58	+	−
Betaine	3.26	+	−
Serine	3.86	−	+
Threonine	1.34	+	−
Trans-4-hydroxy-l-proline	2.14, 3.42	+	−
Glutamate	2.1	−	+
Allantoin	6.05	−	+
NADP+	6.1	−	+
Histidine	7.22	−	+
Citrulline	1.62	+	−
Mannose	5.18	−	+
Glucuronate	3.54	+	−
Indol-3-lactate	7.74	−	+
5.6- Dihydrothymine	2.78	+	−
Cellobiose	3.34	+	−
Arginine	3.26	+	−

## Data Availability

The data used to support the findings of this study are included within the article.
